# Dupilumab-resistant Netherton syndrome associated with a novel SPINK5 variant: c.575A>G (p.Asn192Ser)^؆^

**DOI:** 10.1016/j.abd.2026.501348

**Published:** 2026-04-22

**Authors:** Qingyuan Zhou, Changchun Wang, Qian Jiang, Ruili Jiang, Zilu Qu, Liuqing Chen

**Affiliations:** a Department of Dermatology, Wuhan Hospital of Traditional Chinese and Western Medicine, Tongji Medical College, Huazhong University of Science and Technology, Wuhan, China; b Department of Dermatology, Wuhan No.1 Hospital, Wuhan, China; c Hubei Province Key Laboratory of Skin Infection and Immunity, Wuhan, China; d Department of Dermatology, The Third People's Hospital of Chengdu, Chengdu, Sichuan, China

Dear Editor,

Netherton Syndrome (NS; OMIM #256500) is a rare autosomal recessive disorder caused by pathogenic variants in SPINK5, encoding Lymphoepithelial Kazal-type-related Inhibitor (LEKTI), a protein essential for epidermal barrier integrity.^1^ Loss of LEKTI function results in dysregulated protease activity and the hallmark triad of congenital ichthyosiform erythroderma, trichorrhexis invaginata, and severe atopic diathesis. Although dupilumab has shown therapeutic potential in NS, we report a case of severe, dupilumab-resistant NS associated with a novel SPINK5 variant, c.575A>G (p.Asn192Ser).^2^

An 11-year-old Chinese boy presented with a lifelong history of generalized erythroderma and scaling. He had been repeatedly misdiagnosed with infected eczema or pustular psoriasis, showing minimal response to conventional therapies. Physical examination revealed short stature (height 130 cm, -2.2 SDS) with normal cognitive development. Dermatological assessment demonstrated diffuse erythema with fine-to-lamellar scaling over the head, face, trunk, and limbs, with sparse scalp hair (Fig. 1A‒B). Dermoscopy revealed dotted vascular dilatation, scattered scales, and trichorrhexis invaginata (Fig. 2A‒B). Histopathological examination revealed hyperkeratosis, acanthosis, and superficial perivascular lymphocytic infiltration (Fig. 2C). Immunological evaluation showed markedly elevated serum IgE (> 2500 IU/mL), with allergen-specific IgE positivity to multiple food allergens. Notably, only IL-4 was markedly elevated in the lesional skin (71.33 pg/mg), while other key cytokines ‒ including IL-2, IL-6, IL-10, IL-17, TNF-α, and IFN-γ ‒ remained within normal limits both in the lesion and in serum (Fig. 3).

**Figure 1:**
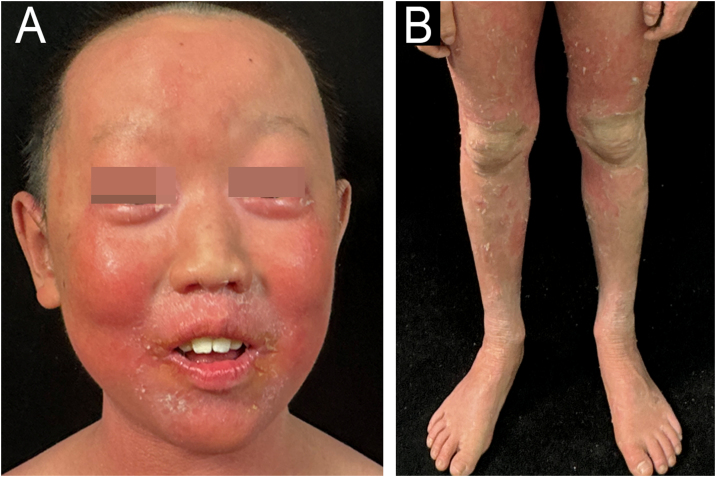
Clinical presentation. (A) Diffuse erythema and fine-to-lamellar scaling over the head and face, accompanied by sparse scalp hair. (B) Diffuse erythema on the limbs.

**Figure 2:**
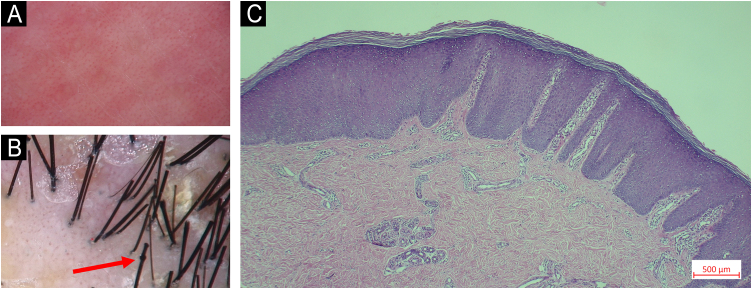
Dermoscopic and histopathological findings. (A) Dermoscopy showing dotted vascular dilatation and scattered scales. (B) Trichoscopy with trichorrhexis invaginata (‘bamboo hair’) on the scalp. (C) Histopathological examination revealing hyperkeratosis, acanthosis, and superficial perivascular lymphocytic infiltration (Hematoxylin & eosin, 200×).

**Figure 3:**
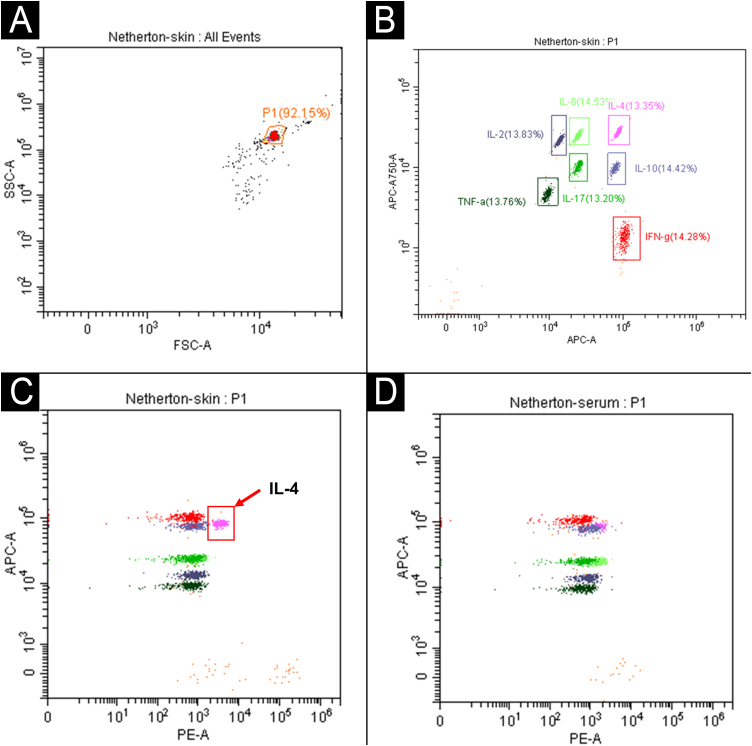
Cytokine profiling reveals tissue-specific elevation of IL-4 in skin lesions of the patient. (A‒B) Schematic of the gating strategy for cytokine bead array analysis. (A) The target bead Population (P1) was gated based on Forward and Side Scatter (FSC-A/SSC-A). (B) Within the P1 population, seven distinct bead subsets were distinguished by APC-A and APC-A750-A signals, each corresponding to a specific cytokine for detection (IL-2, IL-4, IL-6, IL-10, IL-17, TNF-α, and IFN-γ); quantification of each cytokine was ultimately performed via the PE fluorescence channel. (C) Selective elevation of IL-4 in lesional skin (71.33 pg/mg, indicated by a red arrow) against normal reference levels, while other key cytokines ‒ including IL-2, IL-6, IL-10, IL-17, TNF-α, and IFN-γ ‒ were within normal limits. (D) All cytokine concentrations in peripheral blood were within the normal range. Cytokine levels were quantified by flow cytometry using a commercial bead array (Microtech Biotech Co., Ltd.) and analyzed with BD FCAP Array software.

Whole-exome sequencing identified two SPINK5 variants: a previously reported pathogenic nonsense mutation, c.652C>T (p.Arg218*),^3^ and a novel missense variant, c.575A>G (p.Asn192Ser). Sanger sequencing and familial segregation analysis confirmed that the variants were in trans, consistent with a compound heterozygous genotype (Fig. 4A‒B). The novel variant was absent from population databases (gnomAD, LOVD) and was predicted deleterious by multiple in silico tools (SIFT: deleterious; PolyPhen-2: probably damaging; CADD score: 33, top 0.1% of predicted deleterious variants). According to the American College of Medical Genetics and Genomics/Association for Molecular Pathology (ACMG/AMP) guidelines, it was classified as “likely pathogenic” (PM2, PM3, PP3). To our knowledge, this is the first report of this variant in NS.

**Figure 4:**
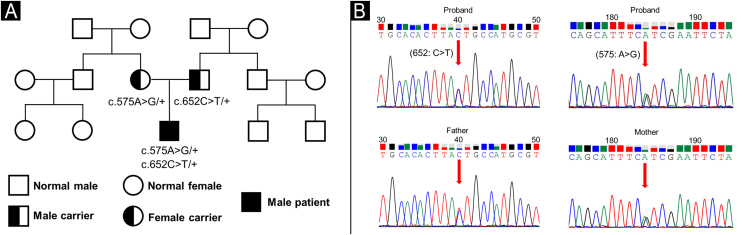
Genetic findings. (A) Family pedigree illustrating the co-segregation of SPINK5 variants. (B) Sanger sequencing of the proband showing compound heterozygosity for c.652C>T (p.Arg218*) and c.575A>G (p.Asn192Ser); each parent heterozygous for one variant.

To further explore the functional impact of these variants, we performed structural modeling of both wild-type and mutant LEKTI using AlphaFold3.^4^ The results revealed that the p.Arg218* nonsense mutation introduces premature termination that truncates the C-terminal domain, likely leading to complete loss of protein function (Fig. 5A‒B). In contrast, the p.Asn192Ser missense mutation does not markedly alter the overall conformation but locally disrupts key hydrogen bonds within a loop region, thereby potentially compromising structural stability (Fig. 5C). Multiple sequence alignment further showed that both residues are highly conserved across species (Fig. 5D), underscoring their critical roles in maintaining LEKTI integrity and function.

**Figure 5:**
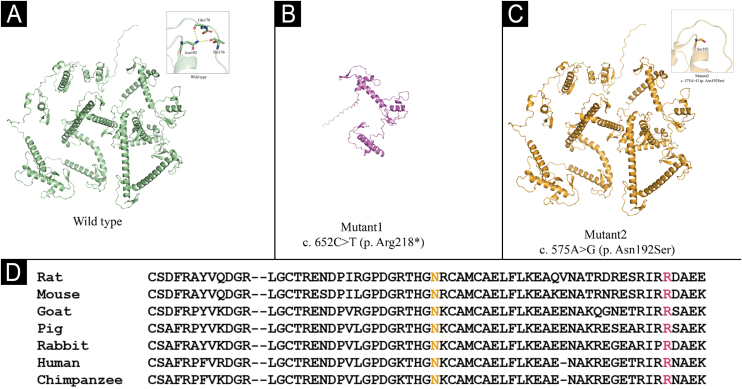
Structural modeling and evolutionary conservation of SPINK5 variants. (A) Wild-type LEKTI protein structure with intact loop conformation (inset). (B) The p.Arg218* truncation mutation results in loss of the C-terminal domain, suggesting complete or severe loss of protein function. (C) The p.Asn192Ser missense mutation disrupts local hydrogen bonds in a loop region (red dashed lines in inset), potentially compromising structural stability. (D) Cross-species sequence alignment shows high conservation of Arg218 and Asn192, indicating their critical roles in maintaining LEKTI structure and function.

As an IL-4 receptor α antagonist, dupilumab has been repeatedly reported to ameliorate Netherton Syndrome (NS) by suppressing Th2-mediated inflammation and improving skin microbiome homeostasis.^5^, ^6^ Despite the patient’s pronounced atopic phenotype and markedly elevated serum IgE, five doses of dupilumab (300 mg each) produced minimal improvement in erythema or pruritus, with facial inflammation even worsened. This lack of clinical efficacy, despite the tissue-specific IL-4 elevation, underscores the immunologic heterogeneity of NS. Although NS is traditionally considered a Th2-polarized disorder, emerging evidence indicates that the erythrodermic subtype exhibits enhanced type I interferon activity, with IL-17, IL-23, and IL-36 pathways also implicated in its pathogenesis.^7^, ^8^ While a direct causal link between the SPINK5 variants and dupilumab resistance cannot be definitively established, genotype-phenotype correlations are well recognized, with 5′-end mutations often associated with more severe clinical manifestations.^9^, ^10^ In this patient, both variants localize to the 5′ region, likely resulting in profound LEKTI deficiency and epidermal barrier disruption. We propose that this upstream defect sustains inflammation via persistent antigen exposure and innate immune activation, thereby undermining the efficacy of Th2-targeted therapy and explaining the observed refractory erythroderma.

In summary, we identified and characterized a novel SPINK5 variant, c.575A>G (p.Asn192Ser), upgrading its classification from uncertain significance to “likely pathogenic” based on ACMG/AMP criteria. Beyond expanding the mutational spectrum of NS, this case of dupilumab-resistant NS underscores the importance of integrating genetic analysis, clinical subtype assessment, and cytokine profiling to guide personalized therapy. Importantly, classical atopic features (e.g., elevated IgE) do not necessarily predict response to Th2-targeted therapy. For patients with severe erythrodermic NS and 5′-region SPINK5 mutations, targeting the IL-4/IL-13 pathway alone may yield limited benefit, even with a marked atopic background.

## ORCID ID

Changchun Wang: 0009-0005-8305-2117

Qian Jiang: 0000-0002-4012-1806

Ruili Jiang: 0009-0007-2069-4360

ZiLu Qu: 0000-0003-2694-7158

Liuqing Chen: 0000-0002-8398-4652

## Research data availability

Does not apply.

## Financial support

This research received financial support from Hubei Provincial Key Research and Development Projects (2023BCB132).

## Author's contribution

Qingyuan Zhou: Performed the experiments and data analysis, and wrote the manuscript.

Changchun Wang: Performed the experiments and data analysis, and wrote the manuscript.

Qian Jiang: Critical review of important intellectual content.

Ruili Jiang: Critical review of important intellectual content.

Zilu Qu: Conceptualized the study and designed experiments; Critically reviewed the manuscript.

Liuqing Chen: Conceptualized the study and designed experiments; Critically reviewed the manuscript.

## Conflicts of interest

None declared.
